# The Biomedical Entrepreneurship Skills Development Program for the Advancement of Research Translation: Foundations of Biomedical Startups course, metrics, and impact

**DOI:** 10.1017/cts.2023.25

**Published:** 2023-02-27

**Authors:** Tucker Schweickart, Zachary Hill-Whilton, Sadhana Chitale, Daniel Cobos, Michal Gilon-Yanai, Joy Achuonjei, Gabriel Vizgan, Colleen Gillespie, Gabrielle Gold-von Simson

**Affiliations:** 1 New York University Grossman School of Medicine, New York, NY, USA; 2 University of Kentucky College of Medicine, Lexington, KY, USA; 3 New York University, Grossman School of Medicine NYU Langone Health NY, New York, NY, USA; 4 New York University, Grossman School of Medicine, Clinical Translational Science Institute, New York, NY, USA; 5 Hofstra University, Zucker School of Medicine, Uniondale, NY, USA; 6 SUNY Downstate College of Medicine, Brooklyn, NY, USA; 7 New York University, Grossman School of Medicine, Department of Pediatrics, PI NIDDK R25, New York, NY, USA

**Keywords:** Entrepreneurship, education, commercialization, venture creation, biotechnology, start-up

## Abstract

**Background/Objective:**

A growing number of biomedical doctoral graduates are entering the biotechnology and industry workforce, though most lack training in business practice. Entrepreneurs can benefit from venture creation and commercialization training that is largely absent from standard biomedical educational curricula. The NYU Biomedical Entrepreneurship Educational Program (BEEP) seeks to fill this training gap to prepare and motivate biomedical entrepreneurs to develop an entrepreneurial skill set, thus accelerating the pace of innovation in technology and business ventures.

**Methods:**

The NYU BEEP Model was developed and implemented with funding from NIDDK and NCATS. The program consists of a core introductory course, topic-based interdisciplinary workshops, venture challenges, on-line modules, and mentorship from experts. Here, we evaluate the efficacy of the core, introductory course, “Foundations of Biomedical Startups,” through the use of pre/post-course surveys and free-response answers.

**Results:**

After 2 years, 153 participants (26% doctoral students, 23% post-doctoral PhDs, 20% faculty, 16% research staff, 15% other) have completed the course. Evaluation data show self-assessed knowledge gain in all domains. The percentage of students rating themselves as either “competent” or “on the way to being an expert” in all areas was significantly higher post-course (*P* < 0.05). In each content area, the percentages of participants rating themselves as “very interested” increased post-course. 95% of those surveyed reported the course met its objectives, and 95% reported a higher likelihood of pursuing commercialization of discoveries post-course.

**Conclusion:**

NYU BEEP can serve as a model to develop similar curricula/programs to enhance entrepreneurial activity of early-stage researchers.

## Introduction

Biomedical entrepreneurship is often regarded as the most challenging sector in the world of ventures with a very low success rate due, in part, to the increasingly high cost of bringing a product to market. For drugs, estimates of total average capitalized pre-launch research and development costs can be as high as $4.5 billion [1,2]. Acquiring funding and complying with a demanding FDA timeline is challenging, time-consuming, and resource intensive, with potential roadblocks at each phase of development [3]. Budding entrepreneurs must, therefore, acquire the skill sets necessary to maximize their chances of success in this difficult-to-navigate field [4]. From forming an optimal team to creating a successful sales funnel, scientists and healthcare professionals are generally not well-versed in standard business practices [5].

While there have been several seemingly successful biomedical entrepreneurship programs [6–13], there is a paucity of educational resources in entrepreneurship for STEM individuals [14]. The creation of new biomedical entrepreneurship foundational courses and/or training programs that cross-pollinate ideas and individuals from across campuses, universities, and regions could potentially fill this translational knowledge gap by introducing scientists to relevant vocabulary, business processes, networking opportunities, as well as other integral aspects of biomedical entrepreneurship [15].

The gap between scientific research and application towards healthcare solutions and medical products is substantial and can potentially be addressed by educating healthcare practitioners in standard business practices [4]. Collaboration between healthcare professionals, academic institutions, and biomedical startups is an effective avenue for innovation in the healthcare field [5]. Furthermore, many biomedical ventures have teamed up with universities to gain scientific credibility for the discoveries that they bring to market, allowing academia to serve as a hub for innovation [16]. This collaboration opens the door to preemptive educational intervention, as business-specific education for early-stage researchers will increase the likelihood of success and ultimately bring more life-changing devices and therapeutics to market. At present, very few US medical schools offer entrepreneurship-based courses [17]. As such, the New York University Grossman School of Medicine’s (NYUGSOM) Biomedical Entrepreneurship Educational Program (BEEP) can serve as a model to bolster national curricula in entrepreneurial skills development and preparedness.

NYU’s multifaceted program consists of a core course, “Foundations of Biomedical Startups, An Introduction to Biomedical Entrepreneurship and Venture Creation,” topic-based multidisciplinary workshops (e.g. “Design Thinking” and “Valuation”), and a mentored “Biomedical Venture Challenge.” Each facet of the program is designed to teach the skills needed to achieve entrepreneurial success. Herein, we present the 2-year metrics for the introductory course, “Foundations of Biomedical Startups.”

## Materials and Methods

### Program Development and Evaluation Plan

**Fig. 1.**
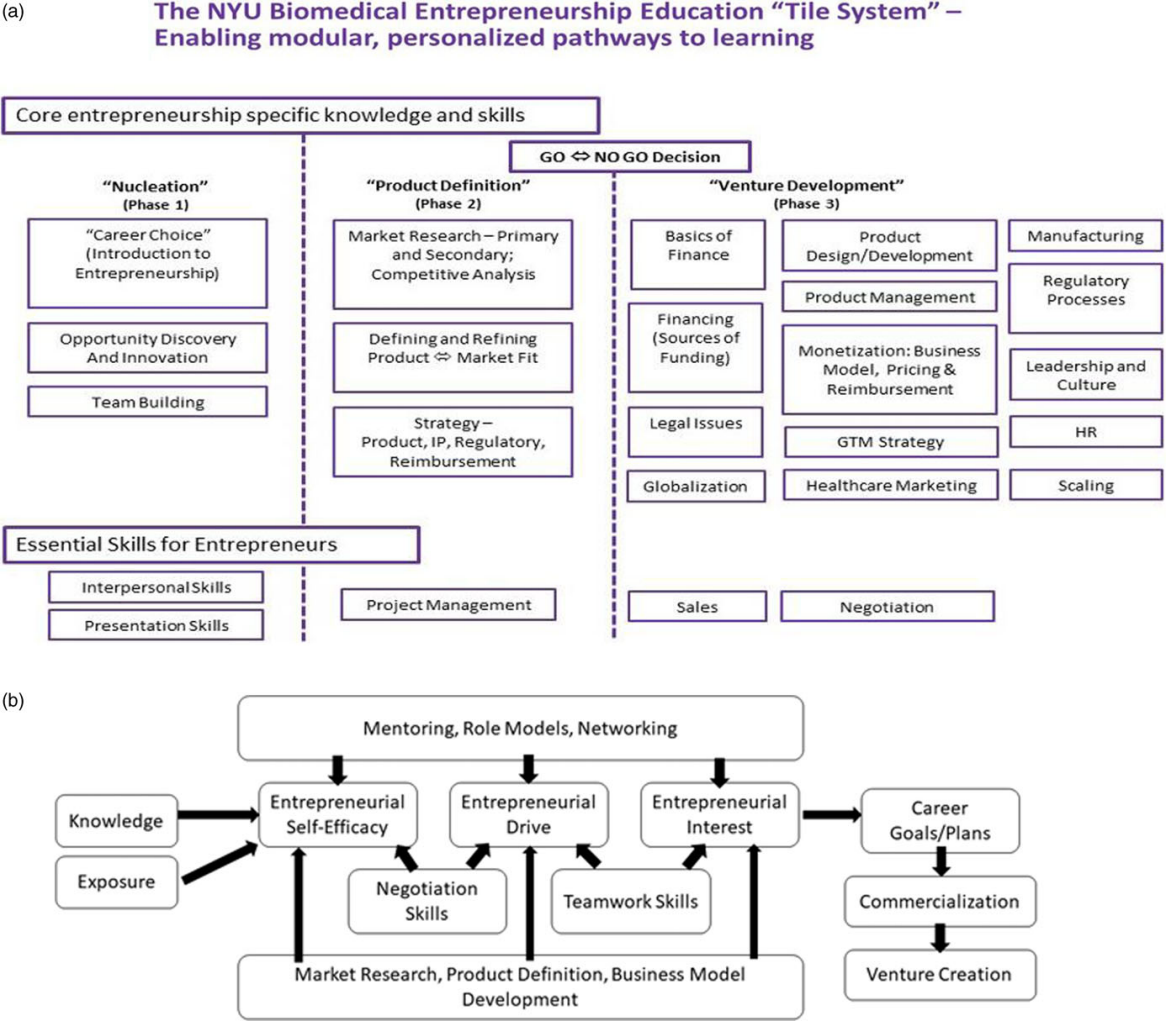
The NYU Biomedical Entrepreneurship Educational Program (BEEP) Development and Framework: (a) The MIT adapted “Tile System” divides the entrepreneurial process into three main stages, “Nucleation,” “Product Definition,” and “Venture Development” and specifies the areas of knowledge and skill required in each [18]. The framework is highly modular and was customized in BEEP for life science entrepreneurs; (b) BEEP Framework and Evaluation Plan Schematic.

The BEEP roadmap was developed using a framework from the Martin Trust Center for MIT Entrepreneurship by an entrepreneurial expert at NYU who previously launched and ran model programs at MIT [18]. During the development stage, our experts extensively surveyed the offerings at other institutions including The University of Michigan (run by Fast Forward Medical Innovation-FFMI) and the Stanford Center for Biodesign. The “Tile System” as depicted in Fig. 1 divides the entrepreneurial process into three main stages: “Nucleation,” “Product Definition,” and “Venture Development,” and specifies the areas of knowledge and skill required in each. The framework is highly modular and was customized in BEEP for life science entrepreneurs. The evaluation plan was based on a logic model that reflected prevailing schematics of entrepreneurial education and best practices for advancing venture creation, particularly in academic medical centers. Studies have consistently demonstrated a positive association between entrepreneurial educational efforts and increases in entrepreneurial self-efficacy, entrepreneurial drive, and entrepreneurial interest and those constructs have, in turn, been linked with longitudinal outcomes like involvement in commercialization and creation of new ventures. Measures of these interim outcomes will help us understand whether the courses and its associated materials and activities are effective for the range of targeted students, including those who do not yet know if they are interested in commercialization, those that think they are but aren’t sure, and those that are sure but do not know how to proceed (Fig. 1) [19].

BEEP was developed and designed for academic entrepreneurs in life sciences to help them better understand the commercial potential and pathway for their research. The program was implemented in 2019, with ongoing improvements based on feedback from participants and the advisory committee as well as new insights into best practices shared by professionals in the field. The development of the program was spearheaded by experts in the field who adapted, for its creation, aspects from various entrepreneurship programs at leading national institutions [18].

A yearlong, two-phase program, the first semester of BEEP provides a general introduction to entrepreneurship in the life sciences industry and teaches basic skills related to the “Nucleation” stage of venture creation, including opportunity discovery and approaches to innovation in the Foundations of Biomedical Startups course (Fig. 1). The second stage of the program is experiential project-based and focuses on “Product Definition” and new venture creation, exploring the commercial potential of new technologies and the search for a viable business model. Upon successful completion of the full program, participants who decide to pursue their projects receive additional support in launching and building their new ventures from the Technology, Opportunities, and Ventures Offices at NYU SOM. Skills development and training is provided in the form of independent lectures and workshops focused on various topics in venture creation. Furthermore, new ventures are supported in commercialization efforts such as Small Business Innovation Research (SBIR) and Small Business Technology Transfer (STTR) grants as well as access to mentors, industry experts, and potential funding opportunities. In this article, we focus on the metrics for the introductory course, “Foundations of Biomedical Startups.”

### Course Description

The current version of the course was first offered in the Fall of 2019 (in-person) and then in the Fall of 2020 (remote). The main focus of the course was on the commercialization of academic discoveries and the entrepreneurial journey of scientists. Through a variety of reading materials, videos, seminars, and case studies presented by experts in the field, participants worked to understand the requirements for launching and building a new venture in the complex and highly regulated life sciences industry and the challenges related to innovation, such as costs, navigating pitfalls and pivots, and globalization, including:

- Understanding initial market research in the landscape of a new technology
- Understanding stakeholders, their needs, and interests
- Understanding workflows, protocols, and emerging new therapeutics in disease
- Defining a high potential application and positioning it correctly
- Assessing the competition
- Developing an intellectual property (IP) strategy and regulatory strategy
- Anticipating financial implications of R&D setbacks and failures
- Planning the effective and efficient utilization of resources etc.

**Fig. 2.**
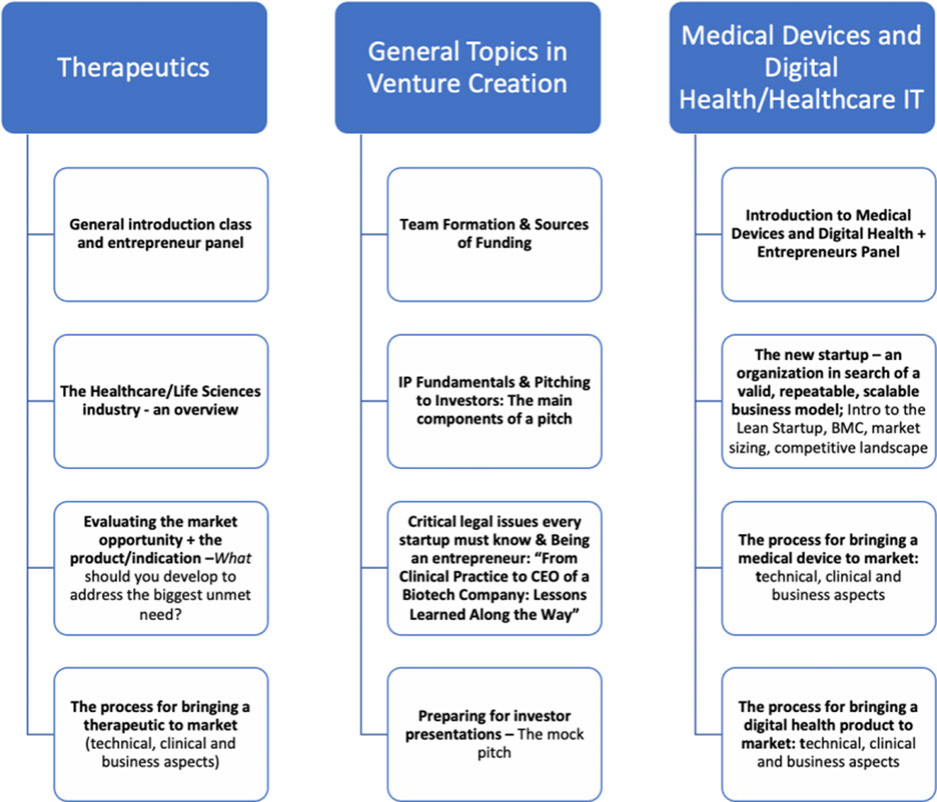
Outline of Syllabus: “Foundations of Biomedical Startups.” The three general areas of focus are Therapeutics, General Topics in Venture Creation, and Medical Devices and Healthcare IT Products.

The course was 12 weeks in duration and was divided into three parts: Therapeutics, General Topics in Venture Creation, and Medical Devices and Healthcare IT Products (Fig. 2).

### Course Enrollment

Though all participants were encouraged to attend all 12 classes, the program was separated into three distinct foci in order to offer a customized learning experience based on the respective interests of each individual student. Students were recruited from all NYU graduate programs, NYUGSOM faculty, staff, and post docs, using science and research focused list-servs. No prerequisites were required, and the program endorsed tuition-free, open enrollment.

### Course Evaluation

#### Pre, midway, and post-course surveys and analysis

Participants received a Qualtrics survey at the beginning, middle, and at the end of the course measuring interest, exposure, knowledge, skills, attitude, and career relevance of course topics. Demographics and other student-level factors and characteristics were collected at the beginning of the course. Students also self-assessed their knowledge, skills, and attitudes towards various competencies (e.g. general business concepts and startup terminology) during the pre- and post-course surveys along a five-point scale. Chi-square tests were run using SPSS to compare changes in responses over time.

#### Lecture ratings

Each lecturer was individually rated in terms of speaker engagement and inspiration as a role model using individual 3-point scales. This format was used to analyze data between lectures particular to each course and between the courses themselves.

#### Free-response text

Free-response questions were included in each survey to add a qualitative measure of course sentiment and to discover opportunities for improvement. The following questions were asked in free-response format:

- Who do you think this course is most ideally suited for?
- Please tell us what your next steps are related to biomedical entrepreneurship.
- What are some of the strengths of the sessions? What are some areas of improvement for the sessions? (asked after first eight lectures and then after final four lectures)
- How do you think the course can get participants to interact with and learn more from each other?
- What has been the main impact of this course on you?
- Which parts of this course do you think could be easily disseminated by sharing the curriculum, online videos, and/or teaching guides?
- Please tell us why you didn’t end up actually attending any of the sessions? (if applicable)
- Overall, please tell us about your main reasons for taking this course - what do you most hope to get out of it?

Data from the survey’s qualitative elements were analyzed to identify key themes around student sentiment, career intentions, and recommendations for improvement and will serve as the basis for more extensive longitudinal surveys.

## Results

### Participant Demographics

**Fig. 3.**
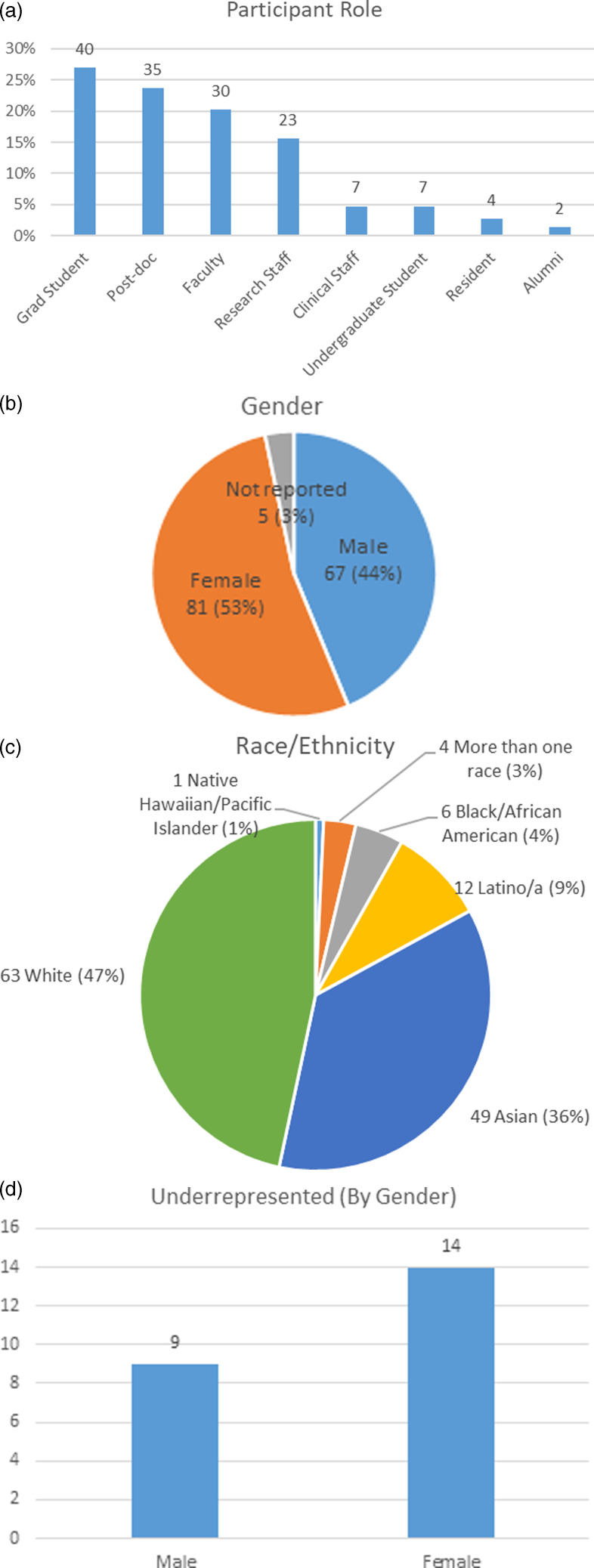
Participant Demographics: (a) Role; (b) Gender; (c) Race/Ethnicity; (d) Underrepresented in science and medicine by gender. Note: the numbers do not necessarily add up to total due to a lack of responses to certain questions.

From the 2019 and 2020 course offerings, a total of 153 participants completed the Foundations of Biomedical Startups course (were present for at least 50% of the lectures). The demographics of the two cohorts have been aggregated to demonstrate that the majority of participants were graduate students (26%), followed by a high participation rate from post-doctoral students (23%), faculty (20%), research staff (16%), and others (15%). Demographic measures included race, ethnicity, gender, and self-perceived representation in medicine. The majority of respondents self-identified as female (53%) and 17% of participants who provided data on race/ethnicity (135) considered themselves to be underrepresented in medicine (“NIH considers the following groups as underrepresented in biomedical research: Individuals from racial and ethnic groups such as Blacks or African Americans, Hispanics or Latinos, American Indians or Alaska Natives, Native Hawaiians, and other Pacific Islanders Individuals with disabilities”) [20]. Among underrepresented groups, the female-male ratio was 1.6:1 (Fig. 3).

### Self-Assessment of Skills

One hundred seventy-one individuals initially expressed interest in the “Foundations of Biomedical Startups” course (69 in 2019; 102 in 2020). of those, 153 registered/participated in the course between August 2019 and December 2020. All participants were asked to complete a self-assessment of their skills prior to the start of the course, 80 of whom completed the pre-course self-assessment. The survey was designed to capture the perceived skills of those interested in biomedical entrepreneurship. At the end of the course, a self-assessment of skills survey was administered to all individuals who had participated in the course (153). Thirty participants completed the post-course survey (17 in 2019; 13 in 2020) or 37.5% of the 80 who provided pre-course data. These survey respondents were more representative of those who regularly attended sessions; the survey was sent to all participants but participants in the last session of each course were given class time to complete the self-assessment and full course evaluation and thus were over-represented among respondents.

**Fig. 4.**
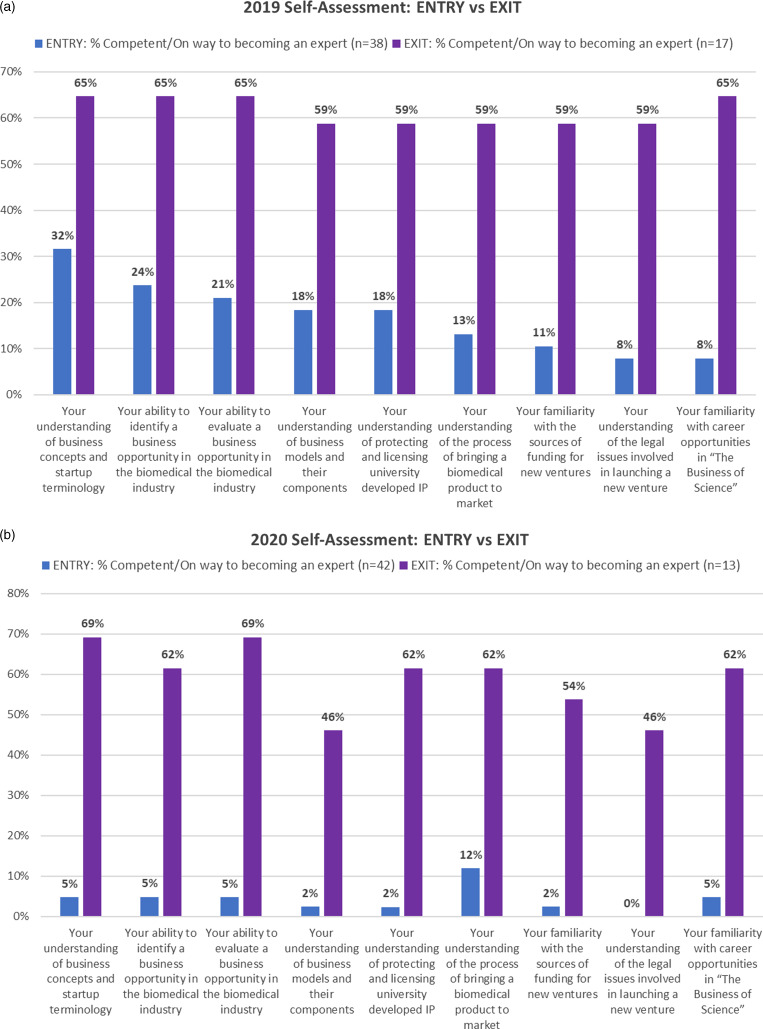
Self-assessment of competence in core course areas: (a) Results of the pre- and post-course surveys for the 2019 “Foundations of Biomedical Startups” course; (b) Results of the pre and post-course surveys for the 2020 “Foundations of Biomedical Startups” course; both achieved statistical significance.

In both cohorts, students self-assessed their competency in several curricular foci before and after course completion on a 4–point scale, ranging from “novice/beginner” to “on the way to being an expert” (Fig. 4). In both 2019 and 2020, the percentage of students rating themselves as either “competent” or “on the way to being an expert” in these areas was found to be significantly higher upon course completion (*P* < 0.05 for all), using a chi-square analysis (Table 1).

**Table 1.** Statistical analysis of 2019 and 2020 pre- and post-course changes in percentage of students self-identifying as competent or on the way to becoming an expert in various curricular domains

	2019 Course Surveys (*n* = 38 pre + 17 post = 55)				2020 Course Surveys (*n* = 42 pre + 13 post = 55)			
	Pre %	Post %	Chi square value	*P* value	Pre %	Post %	Chi square value	*P* value
Your understanding of business concepts and startup terminology	32	65	5.298	0.045	5	69	25.788	< 0.00001
Your ability to identify a business opportunity in the biomedical industry	24	65	8.541	0.009	5	62	21.000	< 0.00001
Your ability to evaluate a business opportunity in the biomedical industry	21	65	9.899	0.005	5	69	25.788	< 0.00001
Your understanding of business models and their components	18	59	8.978	0.007	2	46	16.718	0.0003
Your understanding of protecting and licensing university developed IP	18	59	8.978	0.007	2	62	25.385	< 0.00001
Your understanding of the process of bringing a biomedical product to market	13	59	12.349	0.001	12	62	13.549	0.000942
Your familiarity with the sources of funding for new ventures	11	59	14.439	0.001	2	54	20.669	< 0.00001
Your understanding of the legal issues involved in launching a new venture	8	59	16.878	< 0.0001	0	46	N/A^*^	< 0.0001
Your familiarity with career opportunities in “The Business of Science”	8	65	18.448	< 0.0001	5	62	21.512	< 0.00001

* n was 0 for one category.

### Post-Course Evaluation

Twenty-seven individuals completed both pre- and post-course surveys (30 completed the post-course survey), allowing us to compare self-reported interest in biomedical entrepreneurship topics prior to and then after completing the course and to provide a summary of participants’ views on the degree to which the course met its core objectives. The post-course survey, as noted, was sent to all course participants on the last day of the course but time was set aside during that last session for those in attendance to complete, and so participants who attended the final session (largely those who were present at almost all sessions) are over-represented.

**Fig. 5.**
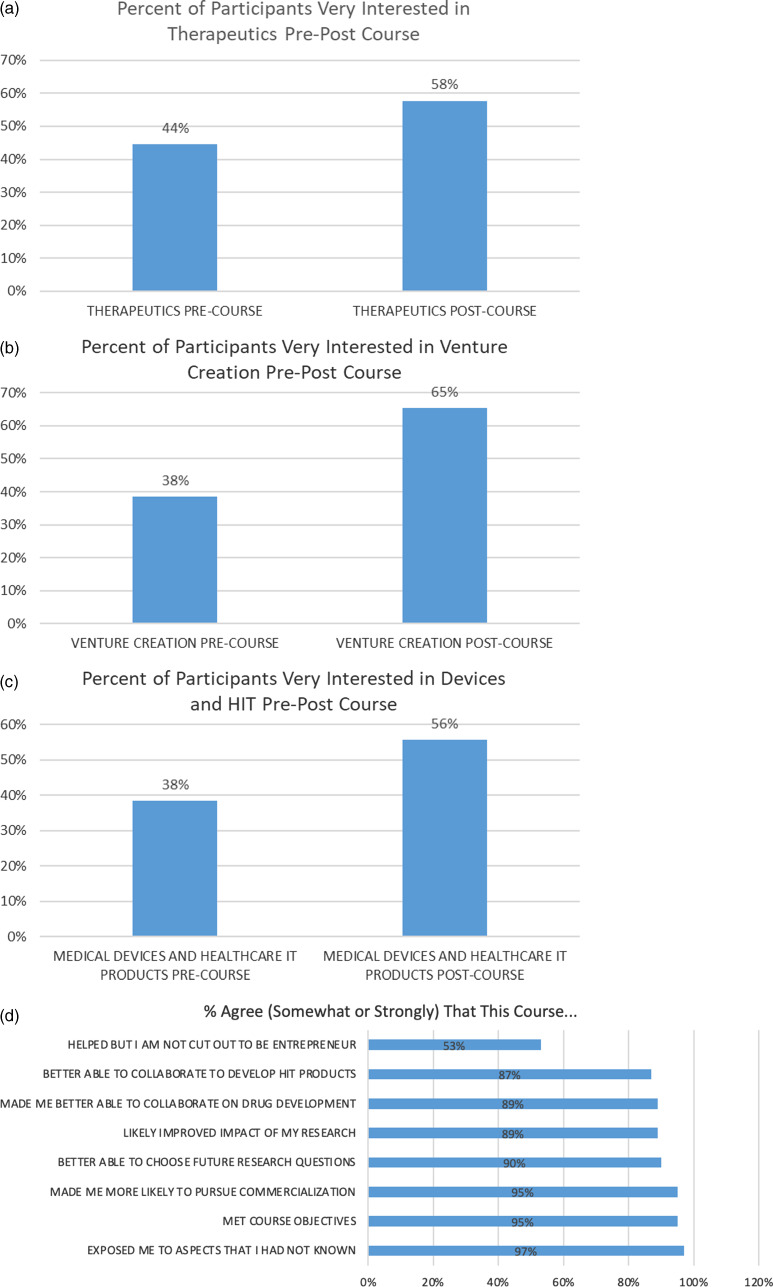
Participant interest per section: (a) 44% of participants at the end of the course reported being very interested in therapeutics before versus 58% after the course; (b) 38% of students reported being very interested in venture creation before versus 65% after the course; (c) 38% of participants reported being very interested in medical devices and healthcare IT before versus 56% after the course; (d) Aggregated data for participants who somewhat or strongly agree that the course met each of its eight objectives.

In the final evaluation, course participants were also asked to rate their level of interest in the three main course content areas (therapeutics, venture creation, and devices) on a 3-point scale before and after having participated in the course (a retrospective pre-post assessment) (n = 27 who answered this item out of the 30 in total who completed the post-survey). For each content area, more participants rated themselves as very interested *after* the course then they had when reflecting back on how interested they were *before* the course (Fig. 5). Using McNemar’s chi-square test for comparing paired proportions (participants serving as their own controls); interest in venture creation trended toward significance. Participants rating themselves as very interested in therapeutics increased by 14% [*X* ^2^ (1, *N* = 27) = 1.29, *P* = 0.26], while those rating themselves as very interested in venture creation increased by 27% [*X* ^2^ (1, *N* = 27) = 3.27, *P* = 0.07], and those very interested in devices increased by 18% [*X* ^2^ (1, *N* = 27) = 1.92, *P* = 0.17].

Students also reported post-course impacts on personal entrepreneurial trajectories, partially as a product of personal ability, motivation, and course quality. A large portion of respondents somewhat or strongly agreed that the course fulfilled each of the given objectives (Fig. 5).

Students’ mean ratings of the degree to which the course met its core objectives did not differ significantly by in-person (Fall 2019) or remote (Fall 2020) format. This pattern was demonstrated across all aspects of the course (e.g., the speakers, the reading materials, the specific topics covered) as well as in the amount of growth in knowledge and competence reflected in the pre/post-course self-assessments. While participants did report wishing the 2020 course could have been in person, as well as challenges fully engaging with speakers and each other during remote sessions, the remote course appeared to deliver an educational experience somewhat equivalent to the in-person version (Table 2).

**Table 2.** Summary comparison for 2019 (in-person) and 2020 (remote) courses (post-course). This table shows the statistical analysis between 2019 and 2020 courses based on mean agreement (1 = strongly disagree to 4 = strongly agree)

Domain	2019 *n* = 17	2020 *n* = 13	*P* value (t-test)
This course so far has definitely met the objectives I had when I registered for it	3.59 ± 0.80	3.85 ± 0.36	0.28
Made me more likely to pursue science/technology commercialization	3.35 ± 0.61	3.38 ± 0.74	0.90
Improved the likely impact of my research	3.24 ± 0.66	3.08 ± 0.62	0.50
Will lead me to choose future research questions/topics that will have more applicability	3.47 ± 0.62	3.31 ± 0.72	0.51
Made me better able to collaborate with those seeking to develop drugs	3.24 ± 0.97	3.31 ± 0.72	0.83
Made me better able to collaborate with those seeking to develop devices	3.41 ± 0.87	3.23 ± 0.70	0.54
Made me better able to collaborate with those seeking to develop digital health/HIT products	3.35 ± 0.70	3.15 ± 0.66	0.28
Exposed me to aspects of biomedical startups that I had not known anything about	3.65 ± 0.61	3.92 ± 0.27	0.15

### Lecturer Ratings

Student ratings of speaker engagement and inspiration as role models were used as key indicators of course quality in both 2019 and 2020. Aggregated over the entire course, 83% of all respondents rated the speakers as either somewhat or very engaging, and 82% rated speakers as somewhat or very inspiring.

### Course Relevance and Objectives

Thirty-eight students completed the course relevance section (1 = strongly disagree to 4 = strongly agree). The mean rating for course relevance was 3.89 (±0.26). Forty-one students completed the course objectives section (1 = strongly disagree to 4 = strongly agree). The mean rating for the meeting of course objectives was 3.61 (±0.66). For those same 41 respondents, a mean response of 3.39 (±0.59) indicated that they were more likely to pursue commercialization. Career relevance was assessed in the form of pre-course surveys. Many participants (68%) stated that their goal was ultimately to work for, or create, their own startup or venture capital firm in the biomedical, life science, or pharmaceutical sector. Twenty-four percent reported their career goal was to obtain an academic position.

## Discussion

Implications of this data delineate the need for the implementation of similar programs for entrepreneurial, venture creation, and commercialization training. The development of an entrepreneurial skill set in the scientific community, which at present is lacking, will serve to accelerate therapeutic innovation within the biomedical sector to satisfy unmet needs.

The course was in person in 2019 and remote in 2020. The data show similar results and feedback in both years, indicating that the course was equally effective in both formats. Some participants believed that they were better able to attend sessions in the remote format due to scheduling/location challenges, while others felt the remote access disrupted discussions, making it difficult to form cohesive bonds with their cohorts and mentors. There are obvious benefits to both formats, however, the in-person dialogue is likely more important in an innovative process.

Regarding the demographic makeup of the 2019 and 2020 participants, the professional diversity varied greatly, which invited a wealth of viewpoints during course discussions, particularly involving career development. The broad professional appeal and relevance of this course to 2019 and 2020 participants is testimony to its value across different fields. To attract a diverse participant pool, the aim was to produce a skills and training program that provided for both short-term and long-term financial and/or intellectual benefit [21]. Those who are underrepresented in medicine and science were recruited via outreach through local NYU and NYC groups. 17% of the course enrollees self-identified as underrepresented in science and medicine, prompting further efforts to extend such outreach especially to those with an interest in biomedical entrepreneurship. As mentioned, the course was free and open to all NYU students, faculty, and staff. In addition, the course was held in the evening, once weekly to accommodate varying schedules to optimize attendance [21].

The course was divided into three sections offering content that provided a well-rounded education while following a logical sequence of focus areas. Attendance data showed that some individuals only attended the lectures most applicable to their career goals. Adjustments were made to maximize course accessibility, but attrition issues persisted with 42% of participants successfully attending “most or all” classes. Scheduling conflicts appeared to be the highest cited reason when participants reported missing classes. The high attrition rate is something that needs to be addressed going forward, but is consistent with voluntary courses in similar fields [22]. Attrition may be addressed by: granting students credit towards a degree program or a certificate; choosing the right time to deliver the course, as per specific cohort feedback; utilizing a hybrid format to allow for remote attendance by some individuals some of the time; connecting with interested students/groups, such as those at the Schools of Engineering and Business.

Studies show that participant affiliation with an academic institution helped predict, and is associated with, participant inventiveness [23]. This is likely true because institutions that are resourced to directly assist in technological development and innovation, intellectual property protections, and commercialization are best positioned to foster biomedical talent and interest. Therefore, academia and university systems are key components of entrepreneurial success and fostering training and skills development in the field is critical for the success of the process, a process that should ideally start during a student’s early career stages.

Survey respondents in 2019 and 2020 reported that course objectives were met across all measured domains and that they learned and benefited from the course. Moreover, the self-assessment data show that the students believed that they were more competent in specific areas after completing the course; there were marked increases in self-perceived competency. Since this course is introductory in nature and requires no prerequisites, participants were expected to have limited knowledge of the subject matter. However, it was found that those with more experience who also attended the course did so in order to further improve their understanding of biomedical entrepreneurship, thus allowing for cross-pollination of ideas among those with different roles, expertise, and levels of training. This finding is a critical benefit to many introductory courses that expose students to areas of interest and help them gauge their compatibility with those interests. Furthermore, the vast majority of participants stated that the course gave them exposure and training that will impact their research, their likelihood to pursue drug, device, and/or digital health product development, as well as science and technology commercialization.

The data delineate the importance and relevance of entrepreneurial curricula in graduate-level science programs for biomedical researchers and clinicians in academia, industry, and other related fields. As most biomedical PhDs graduates do not enter academia and instead enter into the biomedical workforce, entrepreneurial education is particularly essential to provide skills development and training in venture creation and commercialization [24]. Such skills and training will in turn accelerate scientific translation to create relevant and impactful healthcare solutions. Next steps include longer term follow-up of participants to evaluate their contributions to science and industry, their role in the workforce, and their perceived course/program relevance. Specifically, long-term outcomes include entrepreneurial self-efficacy, drive, and intent, career plans, application of skills in practice, participation in science commercialization, grants, patents, biomedical startups as well as sustained participation in commercialization and venture creation. Efforts will also continue to focus on the recruitment of participants who are underrepresented in science and medicine, reduction of attrition, and dissemination of curricula.
